# Protein kinase STK25 aggravates the severity of non-alcoholic fatty pancreas disease in mice

**DOI:** 10.1530/JOE-17-0018

**Published:** 2017-04-25

**Authors:** Esther Nuñez-Durán, Belén Chanclón, Silva Sütt, Joana Real, Hanns-Ulrich Marschall, Ingrid Wernstedt Asterholm, Emmelie Cansby, Margit Mahlapuu

**Affiliations:** 1 Department of Molecular and Clinical MedicineLundberg Laboratory for Diabetes Research, Institute of Medicine, University of Gothenburg, Sahlgrenska University Hospital, Gothenburg, Sweden; 2 Department of Metabolic PhysiologyInstitute of Neuroscience and Physiology, University of Gothenburg, Gothenburg, Sweden; 3 Department of Molecular and Clinical MedicineWallenberg Laboratory, Institute of Medicine, University of Gothenburg, Sahlgrenska University Hospital, Gothenburg, Sweden

**Keywords:** non-alcoholic fatty pancreas disease, ectopic lipid storage, β-cell dysfunction

## Abstract

Characterising the molecular networks that negatively regulate pancreatic β-cell function is essential for understanding the underlying pathogenesis and developing new treatment strategies for type 2 diabetes. We recently identified serine/threonine protein kinase 25 (STK25) as a critical regulator of ectopic fat storage, meta-inflammation, and fibrosis in liver and skeletal muscle. Here, we assessed the role of STK25 in control of progression of non-alcoholic fatty pancreas disease in the context of chronic exposure to dietary lipids in mice. We found that overexpression of STK25 in high-fat-fed transgenic mice aggravated diet-induced lipid storage in the pancreas compared with that of wild-type controls, which was accompanied by exacerbated pancreatic inflammatory cell infiltration, stellate cell activation, fibrosis and apoptosis. Pancreas of *Stk25* transgenic mice also displayed a marked decrease in islet β/α-cell ratio and alteration in the islet architecture with an increased presence of α-cells within the islet core, whereas islet size remained similar between genotypes. After a continued challenge with a high-fat diet, lower levels of fasting plasma insulin and C-peptide, and higher levels of plasma leptin, were detected in *Stk25* transgenic vs wild-type mice. Furthermore, the glucose-stimulated insulin secretion was impaired in high-fat-fed *Stk25* transgenic mice during glucose tolerance test, in spite of higher net change in blood glucose concentrations compared with wild-type controls, suggesting islet β-cell dysfunction. In summary, this study unravels a role for STK25 in determining the susceptibility to diet-induced non-alcoholic fatty pancreas disease in mice in connection to obesity. Our findings highlight STK25 as a potential drug target for metabolic disease.

## Introduction

Type 2 diabetes, characterised by hyperglycaemia in the context of insulin resistance, is one of the most common metabolic diseases in the world. Insulin production from pancreatic β-cells plays a vital role in maintaining the glucose homeostasis in the body, and β-cell dysfunction is central to the pathogenesis of type 2 diabetes. During the progression of obesity and insulin resistance, pancreatic islets initially increase β-cell mass and overproduce insulin; however, the ability of the β-cell to counteract an increased glucose load is short-lived and eventually pancreatic islets fail, giving rise to hyperglycaemia (Saltiel 2001). Hence, characterisation of the critical molecular pathways that negatively modulate pancreatic β-cell function is imperative for understanding the underlying pathogenesis of type 2 diabetes and identifying new pharmacological approaches to effectively restore glycaemia in diabetic patients.

In the search for novel targets regulating energy metabolism, we recently identified serine/threonine protein kinase 25 (STK25, also referred to as YSK1 or SOK1), a broadly expressed member of the sterile 20 (STE20) kinase superfamily (Thompson & Sahai 2015), as a central regulator of whole-body glucose and insulin homeostasis (Nerstedt *et al.* 2012, Cansby *et al.* 2013, Amrutkar *et al.* 2015*a*,*b*, 2016*a*,*b*, Chursa *et al.* 2017). We found that STK25-overexpressing transgenic mice display impaired systemic glucose tolerance and insulin sensitivity compared with wild-type littermates when both genotypes are fed a high-fat diet (Cansby *et al.* 2013). Reciprocally, *Stk25* knockout mice are protected against high-fat diet-induced whole-body glucose intolerance and insulin resistance compared with their wild-type littermates (Amrutkar *et al.* 2015*a*). Furthermore, we observed a markedly accelerated ectopic lipid accumulation, combined with aggravated inflammatory infiltration and fibrosis, in the liver and skeletal muscle of high-fat-fed *Stk25* transgenic mice compared with wild-type controls (Amrutkar *et al.* 2015*b*, Chursa *et al.* 2017), and the reciprocal effect of reduced diet-induced hepatic and muscle lipid storage was seen with STK25 knockdown (Amrutkar *et al.* 2015*a*). Consistent with these preclinical results, we found a significant positive correlation in human liver biopsies between *STK25* expression and progression of non-alcoholic fatty liver disease (NAFLD) (Amrutkar *et al.* 2016*a*,*b*), and higher *STK25* mRNA levels were detected in the skeletal muscle of type 2 diabetic patients compared with individuals with normal glucose tolerance (Nerstedt *et al.* 2012).

Increasing evidence supports the role of fat accumulation in the pancreas (i.e., pancreatic steatosis) in the development of type 2 diabetes, atherosclerosis, acute pancreatitis and pancreatic cancer (Catanzaro *et al.* 2016). In spite of this high medical relevance and an estimated prevalence between 16 and 35% (Wang *et al.* 2014, Lesmana *et al.* 2015, Zhou *et al.* 2016), the pathogenesis of non-alcoholic fatty pancreas disease (NAFPD) is still largely unknown. Our previous investigations demonstrating a critical role of STK25 in regulation of lipid partitioning and ectopic fat storage in liver and skeletal muscle (Nerstedt *et al.* 2012, Cansby *et al.* 2013, Amrutkar *et al.* 2015*a*,*b*, 2016*a*,*b*, Chursa *et al.* 2017) prompted us to hypothesise that STK25 also controls pancreatic steatosis in the context of chronic exposure to excess amount of dietary lipids. By characterisation of pancreatic morphology and function in *Stk25* transgenic mice and wild-type littermates challenged with a high-fat diet, this study unravels a hitherto unknown role of STK25 in determining the susceptibility to NAFPD.

## Materials and methods

### Animals

*Stk25* transgenic mice were generated and genotyped as described previously (Cansby *et al.* 2013). From the age of 6 weeks, male transgenic mice and wild-type littermates were fed a pelleted high-fat diet (45% kilocalories from fat; D12451; Research Diets, New Brunswick, NJ, USA). At the age of 24 weeks, the mice were killed after 4 h of food withdrawal and blood was collected by heart puncture. Pancreas was weighed and collected for histological analysis (see Histology and immunofluorescence) or snap frozen in liquid nitrogen and stored at –80°C for the analysis of gene expression (see Supplementary Fig. 1 for schematic overview, see section on supplementary data given at the end of this article). All animal experiments were performed after approval from the local Ethics Committee for Animal Studies at the Administrative Court of Appeals in Gothenburg, Sweden, and followed appropriate guidelines.

### Histology and immunofluorescence

Pancreas samples were fixed with 4% formaldehyde in phosphate buffer (Histolab Products, Gothenburg, Sweden), embedded in paraffin and sectioned. Islet area, islet perimeter, the number of islets, islet distribution and the number of vessels were assessed in full pancreatic sections stained with hematoxylin-eosin (H-E; Histolab Products). For immunofluorescence, sections were incubated with primary antibodies for STK25, insulin, glucagon or α-smooth muscle actin (α-SMA), followed by incubation with secondary antibodies (see Supplementary Table 1 for antibody information). The distribution of inflammatory cell infiltrate was assessed, and a total insulitis score was calculated as described previously (Fukuda *et al.* 2008) in all islets present in full pancreatic sections stained with Periodic Acid Schiff (PAS) kit (Sigma-Aldrich). Sections were also stained with Picrosirius Red (Histolab Products) and counterstained with Fast Green (Sigma-Aldrich). Apoptotic cells were detected by TUNEL assay using the Apo-BrdU-IHC *In Situ* DNA Fragmentation Assay kit (BioVision).

Pancreas samples were also embedded in optimal cutting temperature mounting medium (Histolab Products) and frozen in liquid nitrogen followed by cryosectioning and staining with Oil Red O (Eastman Kodak Company, Rochester, NY, USA) or MitoTracker Red (Thermo Fisher Scientific). For immunofluorescence, sections were incubated with primary antibodies for Ly6C, followed by incubation with secondary antibodies (see Supplementary Table 1 for antibody information).

All the quantifications were performed in 4–6 non-consecutive pancreatic sections per mouse using the ImageJ software (ImageJ, v1.47; NIH, Bethesda, MD, USA).

Ultrastructural analysis of pancreas was performed by transmission electron microscopy (TEM; LEO 912AB; Omega; Carl Zeiss NTS) as previously described (Anderberg *et al.* 2013).

### Quantitative real-time PCR

Relative quantification of mRNA was performed as described (Cansby *et al.* 2013) using QuantStudio^TM^ 6 Flex System (Applied Biosystems; see Supplementary Table 2 for custom-designed primer sequences). Relative quantities of target transcripts were calculated from duplicate samples after normalisation of the data against the endogenous control, 18S rRNA (Applied Biosystems).

### Biochemical assays

The fasting plasma insulin, C-peptide, leptin and glucagon levels were assessed using the Ultrasensitive Mouse Insulin ELISA kit (Chrystal Chem Inc., Downers Grove, IL, USA), the Mouse C-Peptide ELISA kit (Chrystal Chem Inc.), the Mouse Leptin ELISA kit (Abcam) and the Mouse Glucagon ELISA kit (Chrystal Chem Inc.), respectively. Pancreas homogenates were analysed using the Triglyceride Quantification Colorimetric kit (BioVision) and the Free Fatty Acid Quantification Colorimetric kit (BioVision).

### Determination of Mitochondrial DNA (MtDNA) content

Total DNA was extracted from pancreas using the EZNA Tissue DNA kit (Omega Bio-Tek). MtDNA content was measured with quantitative real-time PCR using the Power UP™ SYBR Green Master Mix (Applied Biosystems) and QuantStudio^TM^ 6 Flex System.

### Intraperitoneal Glucose Tolerance Test (IPGTT)

Following 4 h of food withdrawal, mice were injected with glucose (1 g/kg; Fresenius Kabi, Bad Homburg, Germany) intraperitoneally at time 0. Blood was taken from the tail tip to determine blood glucose concentrations at 0, 15, 30, 60, 90 and 120 min post-injection, using an Accu-Chek glucometer (Roche Diagnostics). The plasma insulin was assessed at 0, 5, 15 and 30 min, and plasma C-peptide was determined at 0 and 15 min after glucose challenge.

### Insulin secretion assay in isolated pancreatic islets

Pancreatic islets were isolated by collagenase digestion as described before (Ammala *et al.* 1993) and incubated overnight at 37ºC in RPMI 1640 medium (Thermo Fisher Scientific) containing 11 mM glucose. The experiments for insulin secretion were carried out in 12-well plates (ten islets per well) for 60 min in RPMI 1640 medium containing 5.5 or 16.5 mM glucose. The medium was collected, and insulin concentration was determined using the Ultrasensitive Mouse Insulin ELISA kit.

### Statistical analysis

Statistical significance between groups was calculated with an unpaired two-tailed Student *t-*test. *P *< 0.05 was considered statistically significant.

## Results

### Overexpression of STK25 alters the distribution of pancreatic α- and β-cells and reduces the islet β/α-cell ratio without any change in islet size

We found that STK25 protein could be detected by immunofluorescence analysis of pancreatic sections both in islets and in surrounding exocrine tissue of *Stk2*5 transgenic mice as well as corresponding wild-type littermates (Supplementary Fig. 2). The immunofluorescence assessment does not allow to reliable quantify the level of overexpression in transgenic mice; however, our previous investigations by quantitative real-time PCR and western blot revealed that STK25 mRNA and protein abundance are increased by approximately 20- and 6-fold, respectively, in whole pancreatic extracts of high-fat-fed *Stk25* transgenic mice compared with those in wild-type mice (Cansby *et al.* 2013). We also found 25.1 ± 3.2-fold overexpression of *Stk25* mRNA in the pancreatic islets isolated from high-fat-fed *Stk25* transgenic mice compared with that in wild-type mice (Supplementary Fig. 3).

**Figure 1:**
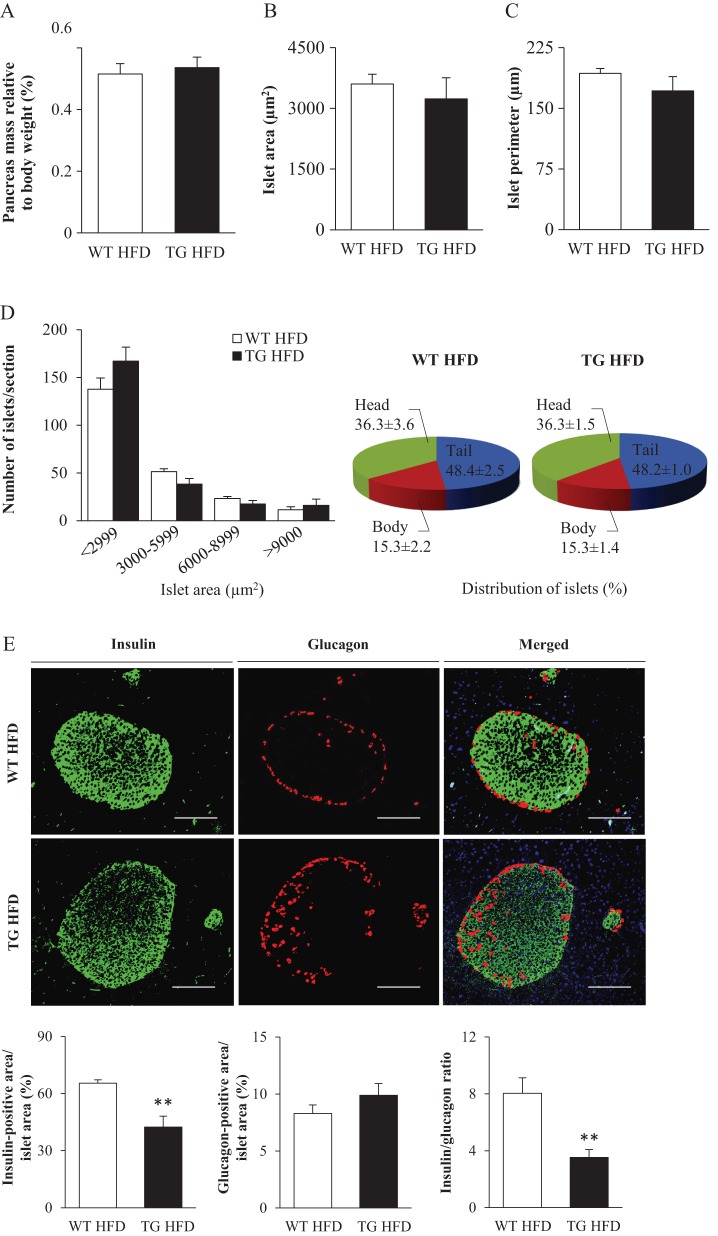
Morphological analysis of pancreatic islets and their cellular composition in high-fat-fed *Stk25* transgenic and wild-type mice. (A) Pancreatic weight. (B–D) The mean area of islets (B), islet perimeter (C) and islet area and regional distribution (D). (E) Representative immunofluorescence images double stained with antibodies for insulin (green) and glucagon (red). Nuclei stained with DAPI are shown in blue in a merged image. Scale bars, 50 µm. Quantification of insulin- and glucagon-positive area, and the islet β/α-cell ratio. Data are mean ± s.e.m. from 5 mice/genotype. **P < 0.01. HFD, high-fat diet; TG, transgenic; WT, wild-type.

We first investigated the overall pancreas morphology in high-fat-fed *Stk25* transgenic and wild-type mice. Our previous studies have shown that *Stk25* transgenic mice challenged with a high-fat diet develop peripheral insulin resistance compared with wild-type littermates (Cansby *et al.* 2013). Diminished insulin sensitivity and a consequent rise in insulin demand are normally compensated by increased islet size (Prentki & Nolan 2006). Notably, we found that the pancreas weight was similar in *Stk25* transgenic vs wild-type mice (Fig. 1A). Moreover, the comprehensive morphometric analysis on H-E-stained pancreatic sections showed that the mean islet area and perimeter, the islet size distribution and the distribution of islets in the head, body and tail region of the pancreas were similar comparing the genotypes (Fig. 1B, C and D), suggesting no compensatory hyperplasia as a result of aggravated insulin resistance in transgenic mice compared with their wild-type controls.

Immunofluorescence staining of the pancreatic sections for insulin and glucagon revealed 1.8 ± 0.2-fold lower insulin-positive area size, with a tendency for higher glucagon-positive area size, in *Stk25* transgenic islets compared with wild-type islets, resulting in 2.6 ± 0.4-fold decrease in islet β/α-cell ratio (Fig. 1E). In wild-type mice, a typical pattern of insulin-producing β-cells clustered in the core of the islet, and a few glucagon-immunoreactive α-cells localised to the periphery of the islet, was observed (Fig. 1E). In contrast, a presence of α-cells within the islet core was frequently seen in transgenic islets (Fig. 1E).

**Figure 2:**
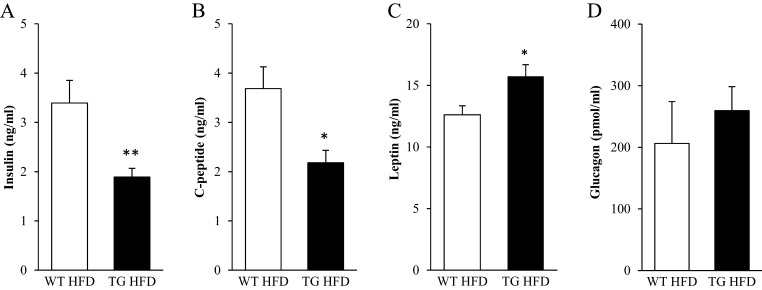
Fasting circulating levels of insulin (A), C-peptide (B), leptin (C) and glucagon (D) in high-fat-fed *Stk25* transgenic and wild-type mice at the time of termination. Data are mean ± s.e.m. from 8 to 11 mice/genotype. *P < 0.05; **P < 0.01. HFD, high-fat diet; TG, transgenic; WT, wild-type.

The mRNA expression of insulin (*Ins1* and *Ins2*) in whole pancreatic extracts did not differ, however, between genotypes, while glucagon mRNA expression (*Gcg*) was 4.6 ± 1.0-fold increased in *Stk25* transgenic mice (Supplementary Fig. 4). At the time of termination, approximately 2-fold lower levels of fasting plasma insulin and C-peptide, and 1.3 ± 0.1-fold higher levels of plasma leptin, were observed in *Stk25* transgenic vs wild-type mice, whereas circulating glucagon levels were similar in both genotypes (Fig. 2).

### STK25 overexpression aggravates diet-induced fat storage in the pancreas

**Figure 3:**
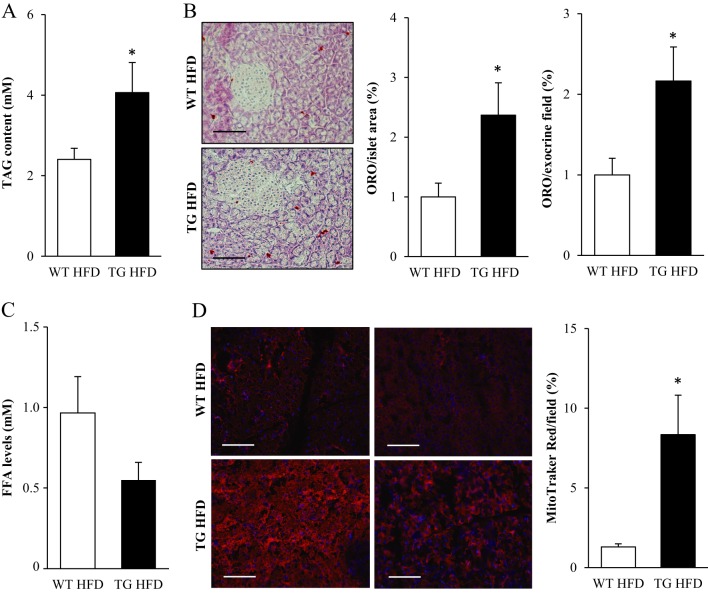
Assessment of fat accumulation and mitochondrial activity in the pancreas of high-fat-fed *Stk25* transgenic and wild-type mice. (A) Triacylglycerol content in pancreatic extracts. (B) Representative images stained with Oil Red O for lipids and counterstained with hematoxylin. Scale bars, 25 µm. Quantification of Oil Red O staining. (C) Free fatty acid levels in pancreatic extracts. (D) Representative images stained with MitoTracker Red; nuclei stained with DAPI (blue). Scale bars, 25 µm. Quantification of MitoTracker Red staining. For A and C, data are mean ± s.e.m. from 11 to 12 mice/genotype; for B and D, data are mean ± s.e.m. from 5–7 mice/genotype. *P < 0.05. FFA, free fatty acid; HFD, high-fat diet; ORO, Oil Red O; TAG, triacylglycerol; TG, transgenic; WT, wild-type.

We next compared lipid accumulation in the pancreatic tissue of high-fat-fed *Stk25* transgenic and wild-type mice. We found that triacylglycerol content extracted from whole pancreas was 1.7 ± 0.3-fold higher in *Stk25* transgenic vs wild-type mice (Fig. 3A). Consistently, histological analysis showed 2.4 ± 0.5- and 2.2 ± 0.4-fold increase in neutral lipids, as judged by Oil Red O staining, in the pancreatic islets and the exocrine pancreatic tissue of *Stk25* transgenic mice, respectively (Fig. 3B). Notably, the content of free fatty acids in the pancreatic extracts was determined to be similar comparing the two genotypes (Fig. 3C).

There are several possible pathways that could underlie the increase in diet-induced lipid deposition in the pancreas of *Stk25* transgenic mice such as enhanced lipid synthesis from one side (input) and repressed mitochondrial fatty acid oxidation on the other side (output) – or a combination of these mechanisms. We found that the pancreatic mRNA expression of the two key enzymes in de novo lipogenesis – acetyl-CoA carboxylase 1 (*Acaca,* also known as *Acc1*) and fatty acid synthase (*Fasn*) – as well as glycerol-3-phosphate acyltransferase (*Gpam,* also known as *Gpat1*), which catalyses the final and only committed step in triacylglycerol synthesis pathway, was similar in *Stk25* transgenic and wild-type mice (Supplementary Fig. 4). To assess mitochondrial activity, we measured the signal for MitoTracker Red, a fluorescent dye that specifically accumulates within respiring mitochondria. Surprisingly, we found that the relative area staining for MitoTracker Red was 6.4 ± 1.9-fold higher, rather than lower, in *Stk25* transgenic vs wild-type pancreas (Fig. 3D). MtDNA copy number in the pancreatic extracts was, however, not altered between genotypes (Supplementary Fig. 5) and imaging by TEM did not reveal any ultrastructural differences comparing the mitochondria in the pancreatic islets of *Stk25* transgenic vs wild-type mice (Supplementary Fig. 6). The mRNA expression of carnitine palmitoyltransferase 1α (*Cpt1α*), an enzyme regulating long-chain fatty acyl-CoA uptake and oxidation in mitochondria, and the components of tricarboxylic acid (TCA) cycle – citrate synthase (*Cs*) and cytochrome c (*Cycs*) – were also similar in *Stk25* transgenic and wild-type pancreas (Supplementary Fig. 4). Similarly, mRNA for fatty acyl-CoA oxidase (*Acox1*), which performs the first step of peroxisomal β-oxidation, was not altered comparing the genotypes (Supplementary Fig. 4).

### Increased inflammatory cell infiltration, fibrosis and apoptosis in the pancreas of *Stk25* transgenic mice

**Figure 4:**
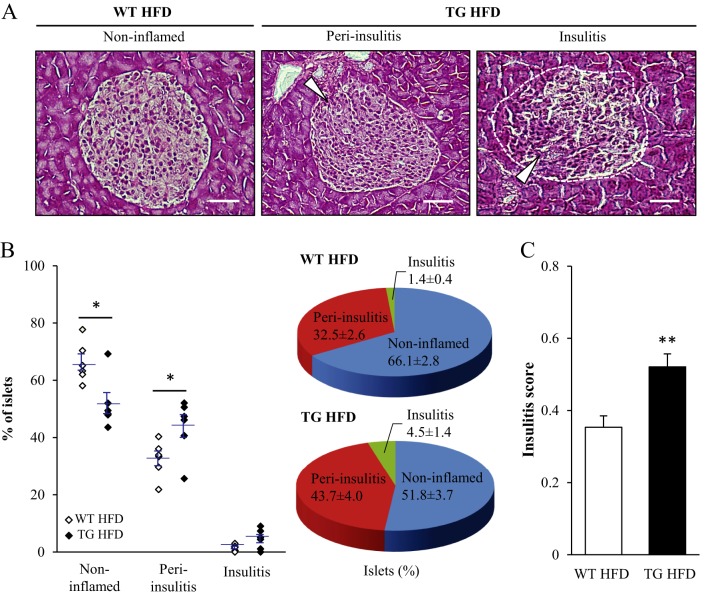
Analysis of inflammatory cell infiltration in the pancreas of high-fat-fed *Stk25* transgenic and wild-type mice. (A) Representative images of non-inflamed islets in wild-type mice, and peri-insulitis and invasive insulitis in *Stk25* transgenic mice. White arrowheads indicate mononuclear cell infiltration. Scale bars, 15 µm. (B) Quantification of non-inflamed islets, peri-insulitis, and insulitis. Results are shown as a dot plot where each point represents one mouse and as a pie chart. (C) The degree of insulitis as assessed using a semi-quantitative scoring system. Data are mean ± s.e.m. from 6 mice/genotype. *P < 0.05; **P < 0.01. HFD, high-fat diet; TG, transgenic; WT, wild-type.

There is a growing recognition that proinflammatory cell infiltration into pancreatic islets in humans and animals with diabetes is involved in the progression of compensated insulin resistance to insulin-dependent type 2 diabetes (Ehses *et al.* 2007, Jourdan *et al.* 2013). We therefore performed histological analysis of the distribution of inflammatory cell infiltrate in pancreatic islets from *Stk25* transgenic and wild-type mice fed a high-fat diet. We found that the percentage of non-inflamed islets was significantly lower in *Stk25* transgenic mice, whereas the percentage of islets with peri-insulitis, defined by the presence of aggregates of mononuclear inflammatory cells surrounding the islets, was significantly higher in *Stk25* transgenic mice compared with wild-type controls (Fig. 4A and B). Furthermore, the relative amount of islets with insulitis, defined by the presence of inflammatory infiltrate within the islets, was also increased in *Stk25* transgenic mice, although this difference did not reach statistical significance (*P* = 0.07; Fig. 4A and B). The total insulitis score based on semi-quantitative grading of non-inflamed islets (score 0), peri-insulitis (score 1) and insulitis (score 2) was 1.5 ± 0.1-fold higher in *Stk25* transgenic vs wild-type mice (Fig. 4C).

Macrophages have been demonstrated to be the most frequent immune cells in the insulitic type 2 diabetic pancreases (Boni-Schnetzler *et al.* 2008, Lundberg *et al.* 2017). Notably, we have previously shown that the liver of *Stk25* transgenic mice fed a methionine- and choline-deficient (MCD) diet displays markedly enhanced staining for the marker of activated macrophages Ly6C compared with corresponding wild-type livers (Amrutkar *et al.* 2016*a*). Consistently, here we found that Ly6C-positive area was augmented by 1.8 ± 0.2-fold in pancreatic islets of *Stk25* transgenic mice compared with corresponding wild-type mice (Supplementary Fig. 7).

**Figure 5:**
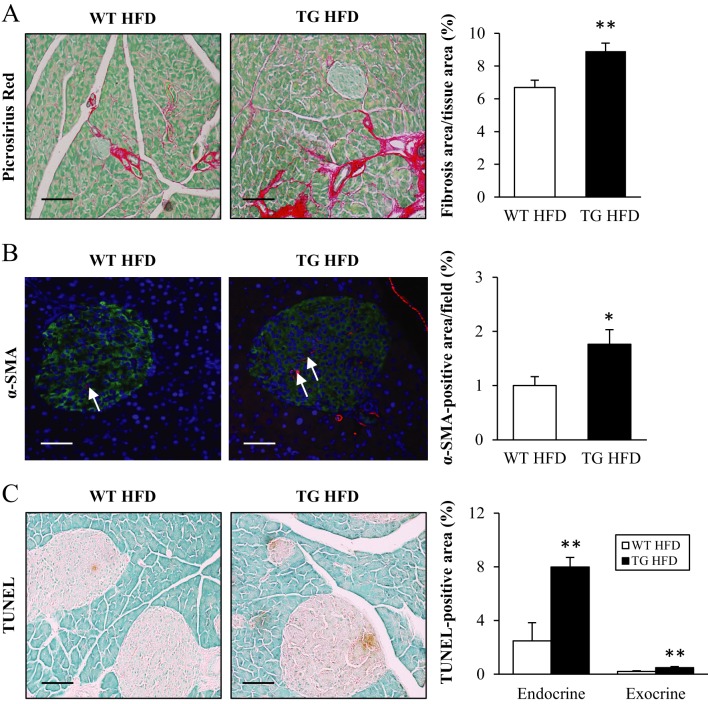
Assessment of fibrosis, PSC activation, and apoptosis in the pancreas of high-fat-fed *Stk25* transgenic and wild-type mice. (A) Representative images stained with Picrosirius Red and counterstained with Fast Green. Quantification of Picrosirius Red staining. (B) Representative images stained with antibodies for α-SMA; nuclei stained with DAPI (blue). White arrows indicate α-SMA-positive cells within the islet. Quantification of α-SMA staining. (C) Representative images stained with TUNEL. Quantification of TUNEL-positive staining. Scale bars, 25 µm. Data are mean ± s.e.m. from 5–8 mice/genotype. *P < 0.05; **P ≤ 0.01. HFD, high-fat diet; TG, transgenic; WT, wild-type.

To assess the degree of fibrosis, the pancreatic sections were stained with Picrosirius Red for collagen fibres. The Picrosirius Red signal was 1.3 ± 0.1-fold higher in *Stk25* transgenic pancreas, with staining being apparent both outside the islets, within the islets and at the islet boundary (Fig. 5A). Furthermore, α-SMA immunohistochemistry was performed to quantify the number of activated pancreatic stellate cells (PSCs), the putative cells responsible for fibrosis in the pancreas. In consistent with Picrosirius Red staining, the α-SMA signal was increased by 1.8 ± 0.3-fold in pancreatic sections from *Stk25* transgenic mice (Fig. 5B). PSCs are also considered to regulate the angiogenesis (Masamune *et al.* 2008, Erkan *et al.* 2009). However, no difference in vessel structure/counts was observed comparing the histological sections from both genotypes (Supplementary Fig. 8).

Lipid overload in the β-cells is known to correlate with programmed cell death in both animal models and humans (Pick *et al.* 1998, Butler *et al.* 2003). We therefore examined *in situ* apoptosis by TUNEL staining that identifies DNA fragmentation within apoptotic cells. TUNEL-positive area was increased 3.0 ± 0.3- and 2.4 ± 0.4-fold in the pancreatic islets and the exocrine pancreatic tissue, respectively, in *Stk25* transgenic mice compared with wild-type littermates (Fig. 5C).

### Overexpression of STK25 represses glucose-stimulated insulin secretion *in vivo*

**Figure 6:**
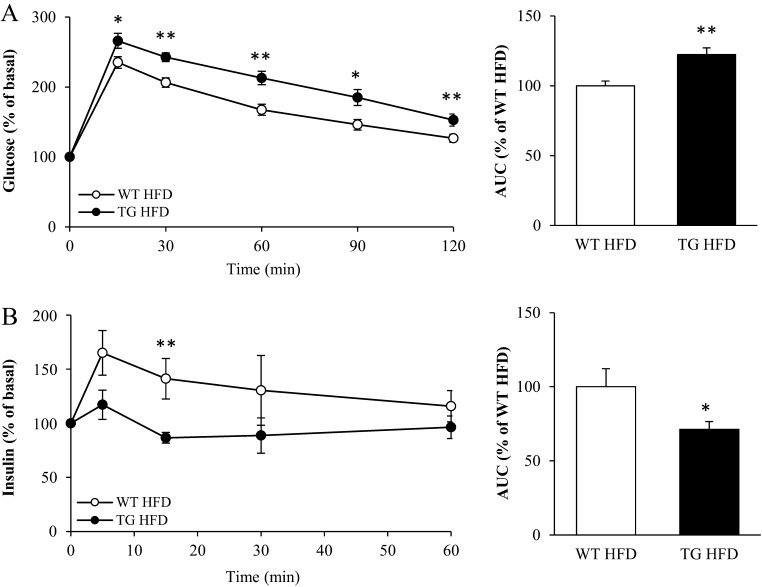
*In vivo* glucose tolerance and glucose-stimulated insulin secretion in high-fat-fed *Stk25* transgenic and wild-type mice. (A) Changes in blood glucose levels following an intraperitoneal injection of glucose. (B) Changes in plasma insulin levels during the experiment shown in (A). Data are mean ± s.e.m. from 9 to 10 mice/genotype. *P ≤ 0.05; **P < 0.01. HFD, high-fat diet; TG, transgenic; WT, wild-type.

To study whether the aggravated fat infiltration, inflammation and fibrosis in the pancreas of *Stk25* transgenic mice leads to an impairment of the *in vivo* glucose-stimulated insulin release, we performed IPGTT in high-fat-fed mice. We found that blood glucose levels were higher in *Stk25* transgenic mice compared with wild-type littermates over the whole time course of the IPGTT (the area under the curve (AUC) for glucose was 22.4 ± 4.8% higher for *Stk25* transgenic than for wild-type mice; Fig. 6A), which is consistent with our previous observation based on a different cohort of mice (Cansby *et al.* 2013). Notably, in spite of higher net change in plasma glucose levels in *Stk25* transgenic mice during IPGTT, the concomitant plasma insulin levels remained lower compared with wild-type littermates (the AUC for insulin was 28.7 ± 5.2% lower for *Stk25* transgenic than for wild-type mice; Fig. 6B), suggesting a relative impairment in glucose-stimulated insulin secretion. The C-peptide/insulin ratio, a marker of insulin clearance (Poy *et al.* 2002, Tamaki *et al.* 2013), was similar comparing the two genotypes at basal conditions as well as at 15 min of glucose challenge (Supplementary Fig. 9).

To evaluate the effect of glucose on insulin secretion from isolated pancreatic islets, we incubated the islets from high-fat-fed mice *in vitro* with 5.5 or 16.5 mM glucose. However, there was no difference in insulin secretion comparing the islets of *Stk25* transgenic vs wild-type mice (Supplementary Fig. 10).

## Discussion

Recent evidence suggests that obesity-related fat accumulation within the pancreas (i.e. NAFPD) is associated with pancreatic inflammation and PSC-mediated fibrogenic processes, and leads to β-cell dysfunction (Smits & van Geenen 2011, Catanzaro *et al.* 2016). In this present study, we unravelled a role for protein kinase STK25 in determining the susceptibility to diet-induced NAFPD in mice by aggravation of pancreatic fat accumulation, inflammatory cell infiltration, PSC activation and fibrosis. Consistent with these morphological alterations in the pancreas, we showed that high-fat-fed STK25-overexpressing transgenic mice had impaired glucose-stimulated insulin release *in vivo* compared with high-fat-fed wild-type controls, which likely contributes to the previously reported systemic glucose intolerance (Cansby *et al.* 2013).

Interestingly, we observed that *Stk25* transgenic mice displayed alterations in islet architecture. Specifically, we noted increased presence of glucagon-producing α-cells in the islet core of transgenic mice, in contrast to the typical arrangement of centrally located insulin-producing β-cells with a satellite distribution of α-cells in the periphery in wild-type mice. The molecular mechanisms linking STK25 overexpression to changes in islet structure remain unclear at present; however, an abnormal spatial relationship between α- and β-cells is frequently observed in mouse models of diet-induced diabetes (Hinault *et al.* 2011, Kai *et al.* 2013, Trasino *et al.* 2015) and it is thought to contribute to β-cell dysfunction by interfering with coordination of β-cell oscillatory activity throughout the islet (Cabrera *et al.* 2006).

We previously showed that peripheral insulin resistance in high-fat-fed *Stk25* transgenic mice compared to wild-type controls is accompanied by hyperinsulinaemia (Cansby *et al.* 2013), suggesting that pancreatic β-cells could potentially increase insulin production in response to increased insulin demands. However, in this study, we observed that, after prolonged high-fat diet challenge, fasting insulin concentrations were lower in *Stk25* transgenic than in wild-type mice at the time of termination. Furthermore, circulating levels of C-peptide were also decreased in *Stk25* transgenic mice at this time point; because C-peptide has a longer half-life than insulin, it is considered a more sensitive index of insulin secretion than fasting insulin. Consistently, we found no evidence of hyperplasia of pancreatic islets by histological analysis in *Stk25* transgenic vs wild-type mice. Taken together, these data suggest that a compensatory capability of β-cells found in *Stk25* transgenic mice at a younger age is lost after a continued high-fat diet challenge. Multiple factors and signalling pathways have been shown to have an impact on decompensation of β-cells and the subsequent progression into diabetes, and in particular, β-cell apoptosis has emerged as a key event (Donath *et al.* 2005, Bonner-Weir *et al.* 2010, Poitout *et al.* 2010, Ashcroft & Rorsman 2012). Indeed, we observed aggravated apoptosis in *Stk25* transgenic pancreas, which probably contributed to the lost ability to increase insulin secretion in the context of insulin resistance. Interestingly, MST1 (also known as STK4 or KRS2), another member of the STE20 kinase superfamily, was recently also identified as a critical regulator of apoptotic β-cell death (Ardestani *et al.* 2014).

The molecular mechanisms that cause pancreatic β-cell damage in the context of obesity are complex and not fully elucidated. In addition to genetic predisposition (O’Rahilly 2009), the combination of excess free fatty acids and glucose (i.e. glucolipotoxicity (Poitout *et al.* 2010, Kim & Yoon 2011, Leibowitz *et al.* 2011)) is known to lead to β-cell death by apoptosis and to decreased insulin secretion. Notably, *Stk25* transgenic and wild-type mice display comparable levels of body fat and circulating lipids (including free fatty acids, total cholesterol and triacylglycerol) after a dietary challenge (Cansby *et al.* 2013, Amrutkar *et al.* 2015*b*). Moreover, fasting blood glucose levels are similar in *Stk25* transgenic and wild-type mice on a high-fat diet and in age-matched lean chow-fed controls (Cansby *et al.* 2013). Therefore, it is likely that the alterations observed in the pancreas of *Stk25* transgenic mice are related to STK25 overexpression, rather than systemic effects through more severe glucolipotoxicity. However, since endogenous STK25 is present in a wide range of different tissues (Pombo *et al.* 1996, Osada *et al.* 1997, Nerstedt *et al.* 2012, Cansby *et al.* 2013) and *Stk25* transgenic mice display global overexpression of the gene (Cansby *et al.* 2013), this model is not suitable to address whether the pancreatic phenotype is due to a direct cell-autonomous effect of STK25 or secondary to the action of STK25 in other tissues. Interestingly, we failed to detect any impairment of glucose-stimulated insulin secretion *in vitro* in pancreatic islets isolated from *Stk25* transgenic mice, suggesting the potential contribution of systemic factors to regulation of glucose-stimulated insulin release by STK25. Of note, in this study, we observed slightly higher circulating leptin levels in high-fat-fed *Stk25* transgenic vs wild-type mice. Leptin is an energy balance-regulating adipokine (Coppari & Bjorbaek 2012) that also is associated with many autoimmune and inflammatory diseases, and higher serum leptin concentrations have previously been connected to inflammatory processes in the pancreas (Kerem *et al.* 2007). It is therefore possible that increased circulating leptin levels contributed to the more severe pancreatic inflammation observed in *Stk25* transgenic vs wild-type mice.

Our previous studies showed that overexpression of STK25 in liver and skeletal muscle cells represses mitochondrial function and β-oxidation, which contributes to increased intrahepatocellular and intramyocellular lipid storage in the respective tissues (Amrutkar *et al.* 2015*b*, Chursa *et al.* 2017). It is known that maintained mitochondrial function in the pancreas is essential to preserve insulin secretion, and impairments in mitochondrial morphology and function have been shown to decrease insulin secretion, presumably through blunted ATP production (Lu *et al.* 2010). It has also been reported that β-cell apoptosis in obesity is induced via a mitochondria-dependent pathway, which is defined by the loss of mitochondrial transmembrane potential and release of proapoptotic signals to the cytosol (Karaskov *et al.* 2006, Martinez *et al.* 2008, Gurzov & Eizirik 2011, Qi & Xia 2012). Unexpectedly, we found increased, rather than decreased, mitochondrial activity in the pancreas of *Stk25* transgenic mice when assessed by MitoTracker Red staining, suggesting that altered mitochondrial activity in transgenic pancreas is likely a compensatory response to a higher lipid load rather than a causative factor driving increased lipid accumulation in this organ. Consistent with this observation, increased hepatic mitochondrial oxidation has been reported in patients and rodents with NAFLD (Sunny *et al.* 2011, Satapati *et al.* 2012), which is considered to reflect a metabolic adaptation to elevated liver lipid burden to limit further fat deposition. However, it is acknowledged that MitoTracker Red staining is an indirect method to monitor mitochondrial activity, and future studies by directly measuring the mitochondrial oxygen consumption in different tissues are needed to fully understand the role of STK25 in the regulation of mitochondrial bioenergetics.

**Figure 7:**
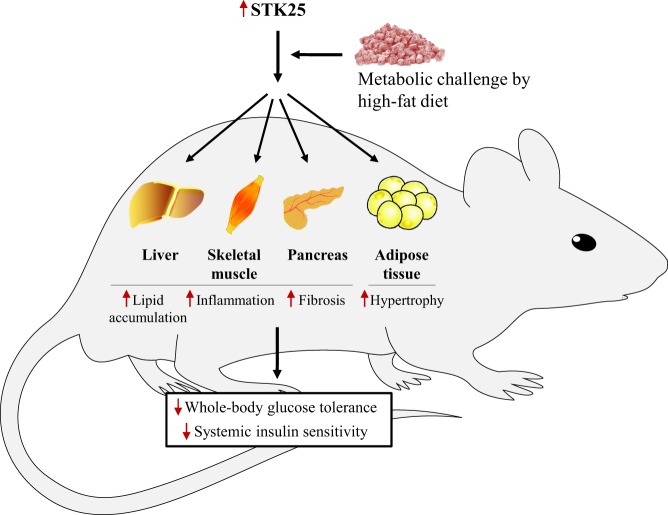
Schematic illustration of metabolic alterations in individual organs and at the whole-body level in *Stk25* transgenic and wild-type mice fed a high-fat diet. Up- or downregulation is indicated by red arrows. The figure combines the results of this study with previously published results (Cansby *et al.* 2013, Amrutkar *et al*. 2015*b*, 2016*a*, Chursa *et al.* 2017).

The alterations in the pancreas of *Stk25* transgenic mice are consistent with our previous investigations in liver and skeletal muscle, where STK25 overexpression under conditions of excess dietary fat aggravates lipid deposition, inflammation and fibrosis (Cansby *et al.* 2013, Amrutkar *et al.* 2015*b*, 2016*a*, Chursa *et al.* 2017). Taken together, these findings suggest that STK25 increases the susceptibility to ectopic lipid storage and meta-inflammation in the main tissues prone to diabetic damage under metabolic stress, resulting in whole-body glucose intolerance and insulin resistance (Fig. 7). Notably, we have also shown earlier that white adipocytes from subcutaneous fat deposits of high-fat-fed *Stk25* transgenic mice are larger than those from high-fat-fed wild-type controls (Cansby *et al.* 2013), indicating that lipid accumulation in the pancreas, liver and muscle is not a consequence of impaired fat storage capacity in adipose tissue (Fig. 7).

In this study, we characterised the pancreatic phenotype of *Stk25* transgenic and wild-type mice challenged with a high-fat diet in order to mimic conditions in high-risk individuals, while chow-fed groups have not been examined, which is a limitation of the study. Notably, our previous investigations have shown that liver and skeletal muscle lipid deposition and whole-body insulin sensitivity are not significantly altered comparing *Stk25* transgenic vs wild-type mice fed regular chow diet (Cansby *et al.* 2013, Amrutkar *et al.* 2016*a*, Chursa *et al.* 2017), suggesting that overexpression of STK25 may lead to significant metabolic alterations in mice only after a dietary challenge.

In summary, the findings in the present study, combined with our previous investigations, reveal that protein kinase STK25 is not only important for regulating peripheral insulin resistance upon chronic exposure to dietary lipids, but it also plays a critical role in β-cell functionality under lipotoxic conditions. Thus, pharmacological manipulation of the STK25 pathway might represent a therapeutic strategy, which combines both regulation of the insulinotropic function and insulin sensitivity in the obese state.

## Supplementary Material

Supporting Figure 1

Supporting Figure 2

Supporting Figure 3

Supporting Figure 4

Supporting Figure 5

Supporting Figure 6

Supporting Figure 7

Supporting Figure 8

Supporting Figure 9

Supporting Figure 10

Supporting Table 1

Supporting Table 2

## Declaration of interest

M M is a founder and shareholder of ScandiCure AB. The authors declare that there is no other duality of interest associated with this article.

## Funding

This work was supported by grants from the Swedish Research Council, the Novo Nordisk Foundation, the Swedish Heart and Lung Foundation, the Diabetes Wellness Network Sweden, the Estonian Research Council, the Swedish Diabetes Foundation, the Royal Society of Arts and Sciences in Gothenburg, the M Bergvalls Foundation, the Wiberg Foundation, the Adlerbert Research Foundation, the I Hultman Foundation, the S and E Goljes Foundation, the West Sweden ALF Program, the F Neubergh Foundation, the I-B and A Lundbergs Research Foundation, and the European Foundation for the Study of Diabetes and Novo Nordisk Partnership for Diabetes Research in Europe.

## Author contribution statement

All the authors made substantial contributions to conception and design, acquisition of data, and/or analysis and interpretation of data. All the authors revised the article critically for important intellectual content and approved the final version of the article to be published. M M and E C wrote the manuscript. M M directed the project and is the guarantor of this work.
