# Supplementary material for: Protein kinase STK25 aggravates the severity of non-alcoholic fatty pancreas disease in mice
Source: J Endocrinol. 2017 Apr 25;234(1):15–27. doi: 10.1530/JOE-17-0018 (PMC5510597; doi:10.1530/JOE-17-0018)
Supplement: Supporting Figure 1 [file joe-234-15-s001.pdf]

ESM Figure 1

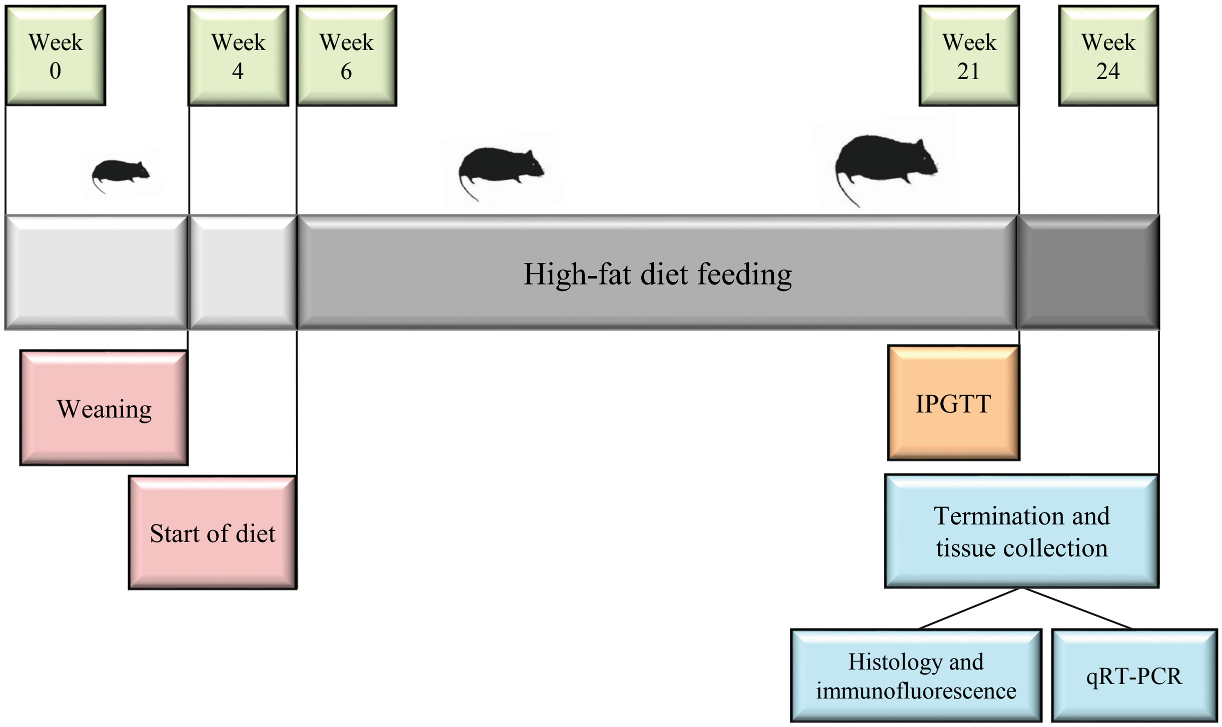

ESM Figure 1. Schematic presentation of measurements performed in high-fat-fed *Stk25* transgenic and wild-type mice. IPGTT, intraperitoneal glucose tolerance test; qRT-PCR, quantitative real-time PCR.
