# Supplementary material for: Protein kinase STK25 aggravates the severity of non-alcoholic fatty pancreas disease in mice
Source: J Endocrinol. 2017 Apr 25;234(1):15–27. doi: 10.1530/JOE-17-0018 (PMC5510597; doi:10.1530/JOE-17-0018)
Supplement: Supporting Figure 2 [file joe-234-15-s002.pdf]

ESM Figure 2

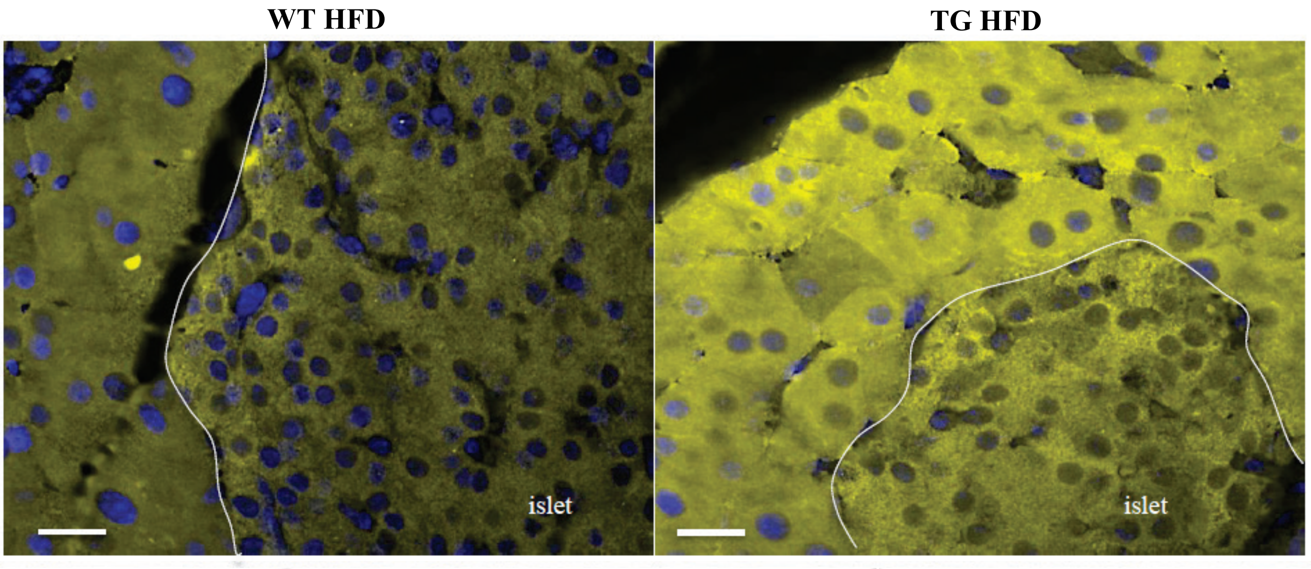

ESM Figure 2. Analysis of STK25 protein abundance in the pancreas of high-fat-fed *Stk25* transgenic and wild-type mice. Representative immunofluorescence images stained with antibody for STK25; nuclei stained with DAPI (blue). Dashed lines denote islet boundaries. Scale bars, 15 µm. HFD, high-fat diet; TG, transgenic; WT, wild-type.
