# Supplementary material for: Protein kinase STK25 aggravates the severity of non-alcoholic fatty pancreas disease in mice
Source: J Endocrinol. 2017 Apr 25;234(1):15–27. doi: 10.1530/JOE-17-0018 (PMC5510597; doi:10.1530/JOE-17-0018)
Supplement: Supporting Figure 3 [file joe-234-15-s003.pdf]

ESM Figure 3

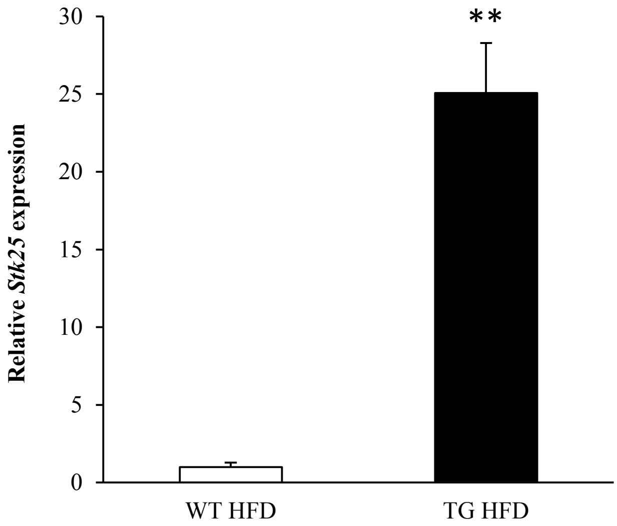

ESM Figure 3. Measurement of mRNA expression in pancreatic islets isolated from high-fat-fed *Stk25* transgenic and wild-type mice. Pancreatic islets were incubated overnight at 37ºC in RPMI 1640 medium containing 11 mM glucose, followed by 60 min incubation in RPMI 1640 medium containing 5.5 mM glucose. Relative mRNA expression was assessed by quantitative real-time PCR. The expression level in wild-type mice is set to 1. Data are mean ± SEM from 4-5 mice per genotype. $^{**}p < 0.001$. HFD, high-fat diet; TG, transgenic; WT, wild-type.
