# Supplementary material for: Protein kinase STK25 aggravates the severity of non-alcoholic fatty pancreas disease in mice
Source: J Endocrinol. 2017 Apr 25;234(1):15–27. doi: 10.1530/JOE-17-0018 (PMC5510597; doi:10.1530/JOE-17-0018)
Supplement: Supporting Figure 4 [file joe-234-15-s004.pdf]

ESM Figure 4

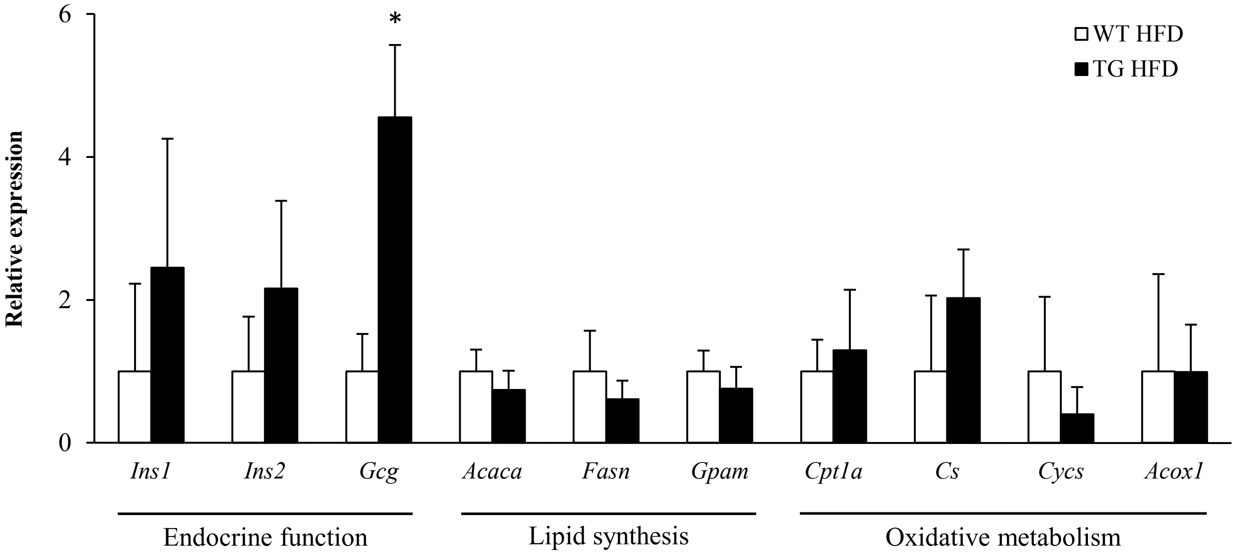

ESM Figure 4. Measurement of mRNA expression in pancreatic extracts of high-fat-fed *Stk25* transgenic and wild-type mice. Relative mRNA expression was assessed by quantitative real-time PCR. The expression level of each gene in wild-type mice is set to 1. The gene functions are indicated at the bottom. Data are mean ± SEM from 7-12 mice per genotype. *$p < 0.05$.
