# Supplementary material for: Protein kinase STK25 aggravates the severity of non-alcoholic fatty pancreas disease in mice
Source: J Endocrinol. 2017 Apr 25;234(1):15–27. doi: 10.1530/JOE-17-0018 (PMC5510597; doi:10.1530/JOE-17-0018)
Supplement: Supporting Figure 5 [file joe-234-15-s005.pdf]

ESM Figure 5

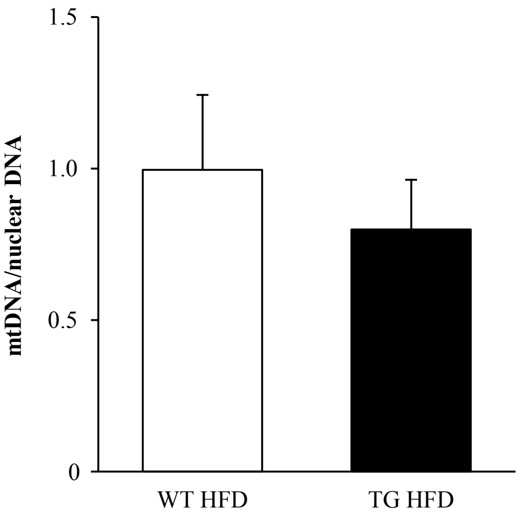

ESM Figure 5. Measurement of mtDNA content in pancreatic extracts of high-fat-fed *Stk25* transgenic and wild-type mice. Relative mtDNA content was calculated as the ratio of a mitochondrial-encoded gene (*Cox1*, forward 5′-ACTATACTACTACTAACAGACCG-3′, reverse 5′-GGTTCTTTTTTTCCGGAGTA-3′) to a nuclear-encoded gene (*Cyclophilin A*, forward 5′-ACACGCCATAATGGCACTGG-3′, reverse 5′-CAGTCTTGGCAGTGCAGAT-3′) DNA levels, determined by quantitative real-time PCR. The mtDNA content in wild-type mice is set to 1. Data are mean ± SEM from 10 mice per genotype. HFD, high-fat diet; TG, transgenic; WT, wild-type.
