# Supplementary material for: Protein kinase STK25 aggravates the severity of non-alcoholic fatty pancreas disease in mice
Source: J Endocrinol. 2017 Apr 25;234(1):15–27. doi: 10.1530/JOE-17-0018 (PMC5510597; doi:10.1530/JOE-17-0018)
Supplement: Supporting Figure 6 [file joe-234-15-s006.pdf]

ESM Figure 6

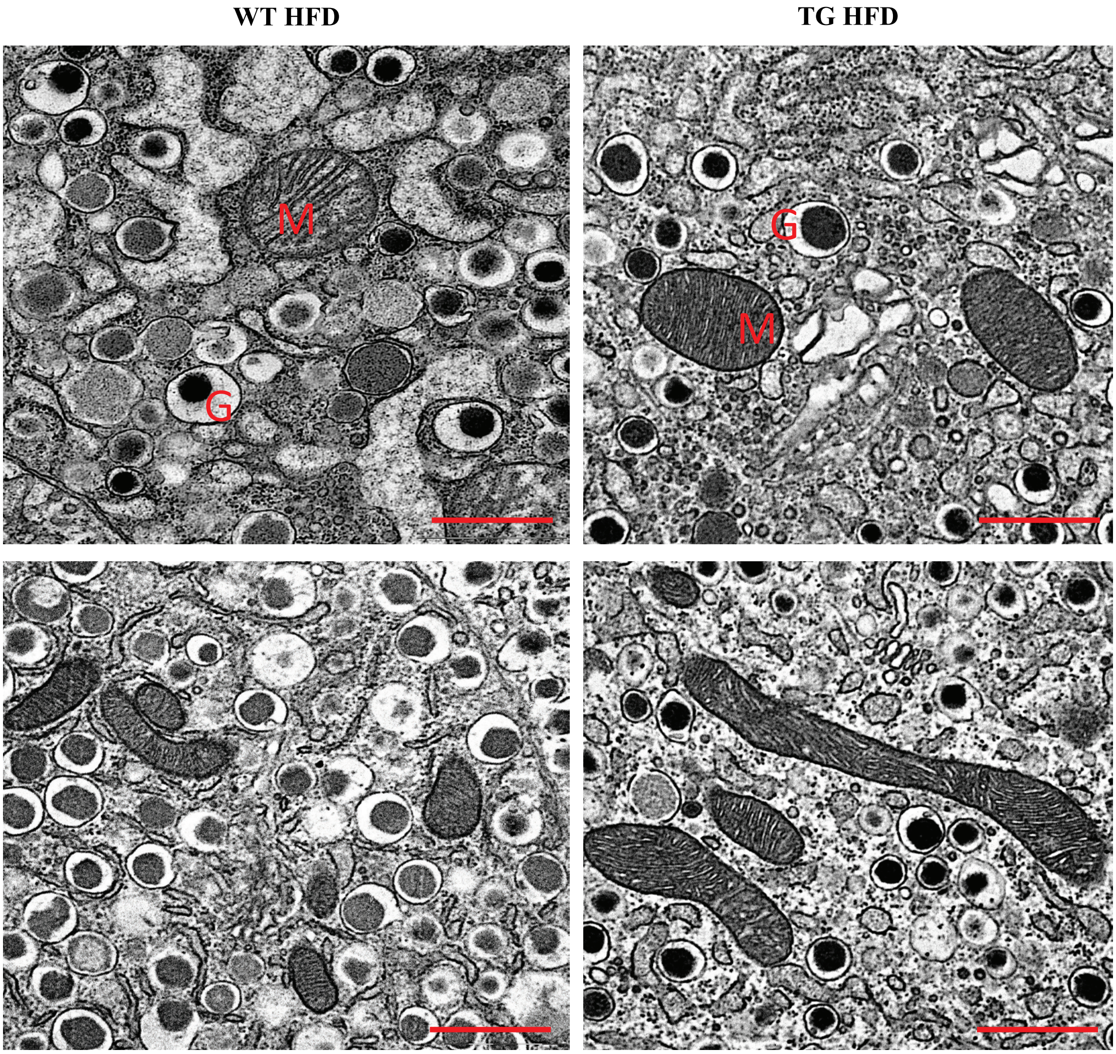

ESM Figure 6. Representative transmission electron microscopy images of the pancreatic islets of high-fat-fed *Stk25* transgenic and wild-type mice. Scale bars, 1 µm. G, insulin granules; HFD, high-fat diet; M, mitochondria; TG, transgenic; WT, wild type.
