# Supplementary material for: Protein kinase STK25 aggravates the severity of non-alcoholic fatty pancreas disease in mice
Source: J Endocrinol. 2017 Apr 25;234(1):15–27. doi: 10.1530/JOE-17-0018 (PMC5510597; doi:10.1530/JOE-17-0018)
Supplement: Supporting Figure 8 [file joe-234-15-s008.pdf]

ESM Figure 8

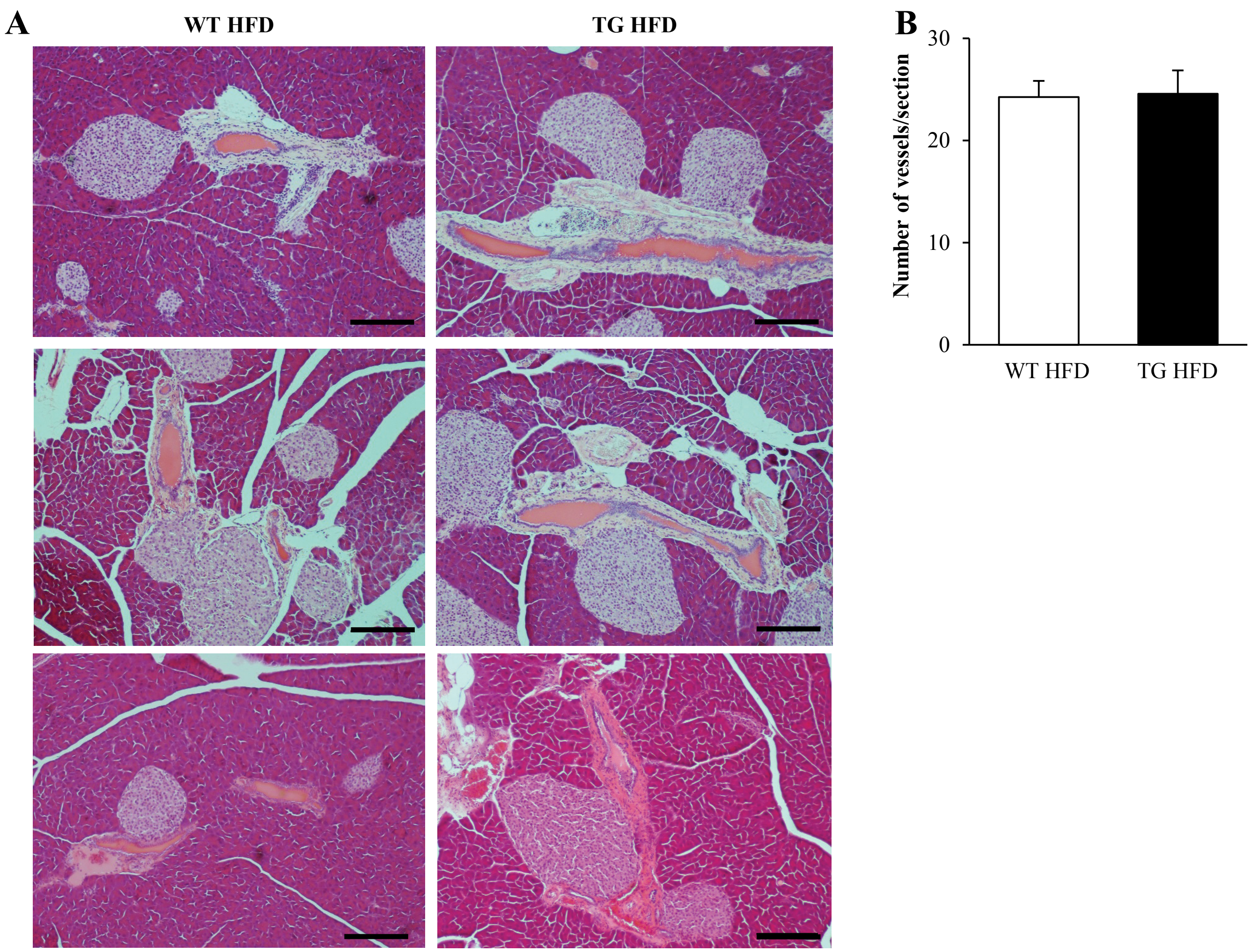

ESM Figure 8. Quantification of the number of vessels in the pancreatic tissue of high-fat-fed *Stk25* transgenic and wild-type mice. *A*: Representative images stained with H-E. Scale bars, 50 µm. *B*: Quantification of the number of vessels. Data are mean ± SEM from 6 mice per genotype. HFD, high-fat diet; TG, transgenic; WT, wild-type.
