# Supplementary material for: Protein kinase STK25 aggravates the severity of non-alcoholic fatty pancreas disease in mice
Source: J Endocrinol. 2017 Apr 25;234(1):15–27. doi: 10.1530/JOE-17-0018 (PMC5510597; doi:10.1530/JOE-17-0018)
Supplement: Supporting Figure 9 [file joe-234-15-s009.pdf]

ESM Figure 9

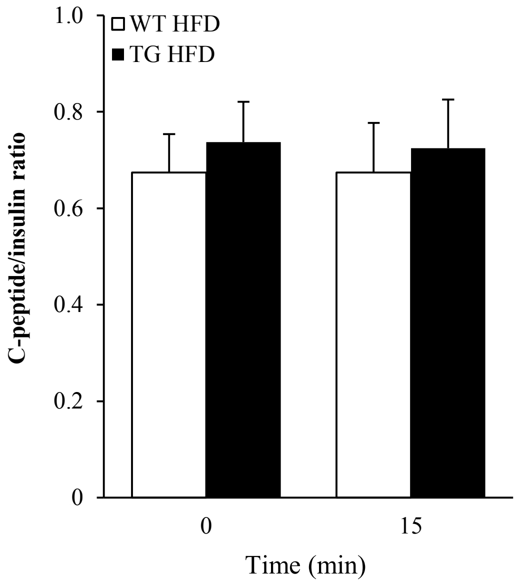

ESM Figure 9. C-peptide/insulin ratio assessed during IPGTT in high-fat-fed *Stk25* transgenic and wild-type mice. The levels were measured in plasma collected during the experiment shown in Fig. 6. Data are mean ± SEM from 12-13 mice per genotype. HFD, high-fat diet; TG, transgenic; WT, wild-type.
