# Supplementary material for: Protein kinase STK25 aggravates the severity of non-alcoholic fatty pancreas disease in mice
Source: J Endocrinol. 2017 Apr 25;234(1):15–27. doi: 10.1530/JOE-17-0018 (PMC5510597; doi:10.1530/JOE-17-0018)
Supplement: Supporting Figure 10 [file joe-234-15-s010.pdf]

ESM Figure 10

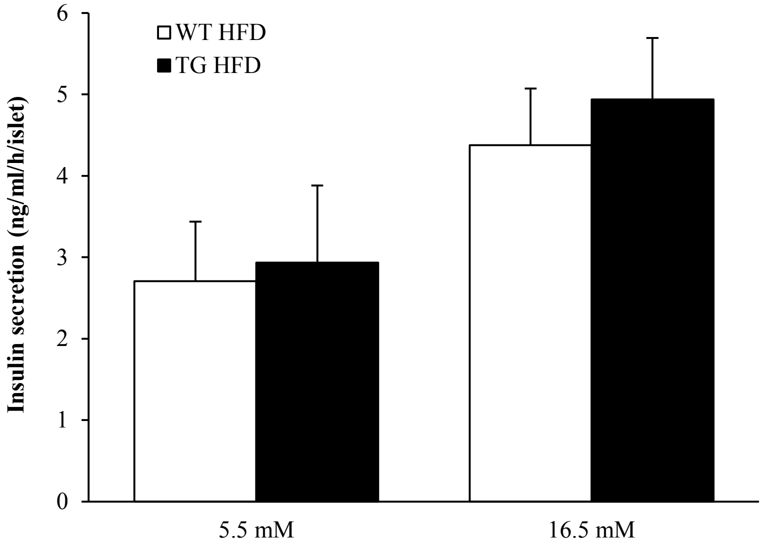

ESM Figure 10. *In vitro* glucose-stimulated insulin secretion from pancreatic islets isolated from high-fat-fed *Stk25* transgenic and wild-type mice. Data are mean ± SEM from 4-5 mice per genotype. HFD, high-fat diet; TG, transgenic; WT, wild-type.
