# Supplementary material for: Protein kinase STK25 aggravates the severity of non-alcoholic fatty pancreas disease in mice
Source: J Endocrinol. 2017 Apr 25;234(1):15–27. doi: 10.1530/JOE-17-0018 (PMC5510597; doi:10.1530/JOE-17-0018)
Supplement: Supporting Table 1 [file joe-234-15-t001.pdf]

ESM Table 1. List of antibodies used for immunofluorescence

**Type**	**Antibody name and catalogue number**	**Working dilution**	**Company**
Primary antibody	anti-glucagon (#G2654)	1:1000	Sigma-Aldrich (St. Louis, MO, USA)
	anti-insulin (#ab7842)	1:800	Abcam (Cambridge, UK)
	anti-α-SMA (#ab5694)	1:50	Abcam
	anti-Ly6C (anti-Gr1; #ab15627)	1:500	Abcam
	anti-STK25 (#NBP1-32670)	1:300	Novus Biologicals (Littleton, CO, USA)
Secondary antibody	donkey anti-rabbit (#A21207)	1:1000	Invitrogen (Carlsbad, California, USA)
	donkey anti-mouse (#A21203)	1:1500	Invitrogen
	goat anti-guinea pig (#A11073)	1:1500	Invitrogen
	goat anti-rat (#A11007)	1:600	Invitrogen
