# Supplementary material for: Protein kinase STK25 aggravates the severity of non-alcoholic fatty pancreas disease in mice
Source: J Endocrinol. 2017 Apr 25;234(1):15–27. doi: 10.1530/JOE-17-0018 (PMC5510597; doi:10.1530/JOE-17-0018)
Supplement: Supporting Table 2 [file joe-234-15-t002.pdf]

ESM Table 2. Sequences of custom-designed primers (forward and reverse) and probes used for quantitative real-time PCR

Gene		Sequence (5’-3’)
Cycs	Forward	GGACCAAATCTCCACGGTCT
	Reverse	CCCCAGGTGATGCCTTTGT
	Probe	AGCCTGGCCTGTCTTCCGCCC
Fasn	Forward	CTTAGCAGAGATCCCGAGACG
	Reverse	GGTCCTTTGAAGTCGAAGAAGAA
	Probe	TGGGCTACAGCATGGTGGGCTG
